# Advances in highly doped upconversion nanoparticles

**DOI:** 10.1038/s41467-018-04813-5

**Published:** 2018-06-20

**Authors:** Shihui Wen, Jiajia Zhou, Kezhi Zheng, Artur Bednarkiewicz, Xiaogang Liu, Dayong Jin

**Affiliations:** 1 Institute for Biomedical Materials & Devices (IBMD), Faculty of Science, University of Technology Sydney, Sydney, NSW 2007 Australia; 2 Department of Chemistry, National University of Singapore, 3 Science Drive 3, Singapore, 117543 Singapore; 3 Institute of Low Temperature and Structure Research, Polish Academy of Sciences, Okólna 2, 50-422 Wroclaw, Poland; 4 Wroclaw Research Center, EIT+, Stablowicka 147, 54-066 Wroclaw, Poland

## Abstract

Lanthanide-doped upconversion nanoparticles (UCNPs) are capable of converting near-infra-red excitation into visible and ultraviolet emission. Their unique optical properties have advanced a broad range of applications, such as fluorescent microscopy, deep-tissue bioimaging, nanomedicine, optogenetics, security labelling and volumetric display. However, the constraint of concentration quenching on upconversion luminescence has hampered the nanoscience community to develop bright UCNPs with a large number of dopants. This review surveys recent advances in developing highly doped UCNPs, highlights the strategies that bypass the concentration quenching effect, and discusses new optical properties as well as emerging applications enabled by these nanoparticles.

## Introduction

Upconversion nanoparticles (UCNPs) are a unique class of optical nanomaterials doped with lanthanide ions featuring a wealth of electronic transitions within the 4*f* electron shells. These nanoparticles can up-convert two or more lower-energy photons into one high-energy photon^1–3^. Over the past decade, this unique anti-Stokes optical property has enabled a broad range of applications, spanning from background-free biological sensing and light-triggered drug delivery to solar energy harvesting and super-resolution microscopy^4–6^. To achieve high upconversion efficiency, it is essential to co-dope sensitizer ions alongside activator ions that have a closely matched intermediate-excited state^7–9^. This doping process requires a rational design that offers optimal interactions of a network of the sensitizer and activator ions, and the upconversion efficiency is highly dependent on the separating distance between the dopants. Therefore, the proper management of the doping concentration in a given nanoparticle will be the deciding factor in leveraging the energy transfer process and ultimately the luminescence performance of the nanoparticle^9–11^.

In stark contrast to quantum dots, UCNPs contain individual and variable absorption and emission centres. Thus, the primary goal to increase the concentration of co-dopants in UCNPs is to directly improve their brightness. However, the constraint of concentration quenching that limits the amount of the dopants usable has been known for years in bulk materials (for example, Nd^3+^-doped YAG laser crystals)^12^, and tuning the luminescence properties has been largely hindered at relatively low-doping concentrations^2,13^. For nanomaterials with a high ratio of surface area to volume, high-doping concentration is likely to induce both cross-relaxation energy loss and energy migration to the surface quenchers. This, in turn, explains the much reduced luminescence quantum yield in upconversion nanomaterials relative to their bulk counterparts^14–16^. Encouragingly, over the past decade, a great deal of research efforts has been devoted to the study of the concentration quenching mechanisms^2,10,17,18^, thereby opening the door to many ground-breaking applications.

In this review, we discuss the phenomenon and underlying mechanism of concentration quenching occurring in UCNPs, review the general and emerging strategies for overcoming the concentration quenching effect, and summarize the impact of highly doped UCNPs on a range of disruptive applications. In particular, we discuss the rational design of heterogeneously doped, multilayered UCNPs that allow us to precisely control the energy migration process and induce cross-relaxation between the dopants for unprecedented optical phenomena. We present the challenges and opportunities of the doping strategies in developing smaller and brighter nanoparticles as well as hybrid materials with synergetic multifunction.

### Concentration quenching

For a very long time, the problem of concentration quenching was the major obstacle that hindered the quest for highly luminescent materials^12,19^. The theory of concentration quenching in inorganic phosphors was introduced in 1954 by Dexter and Schulman, who pointed out that considerable quenching of luminescence in bulk materials arises when the activator concentration reaches 10^−3^–10^−2^ M[19]. Different mechanisms (resonance energy transfer^20^, molecular interactions^21^, and intermolecular photo-induced electron transfer^22^) of concentration quenching in organic dyes have been studied since the early 1980s. The issue has limited the maximum number of fluorophores allowed in dye-doped silica nanoparticles^23^. As a result, the detrimental effect of concentration quenching in luminescent materials imposes a restriction on access to a high level of luminescence intensity, in consequence hindering their further applications.

**Fig. 1:**
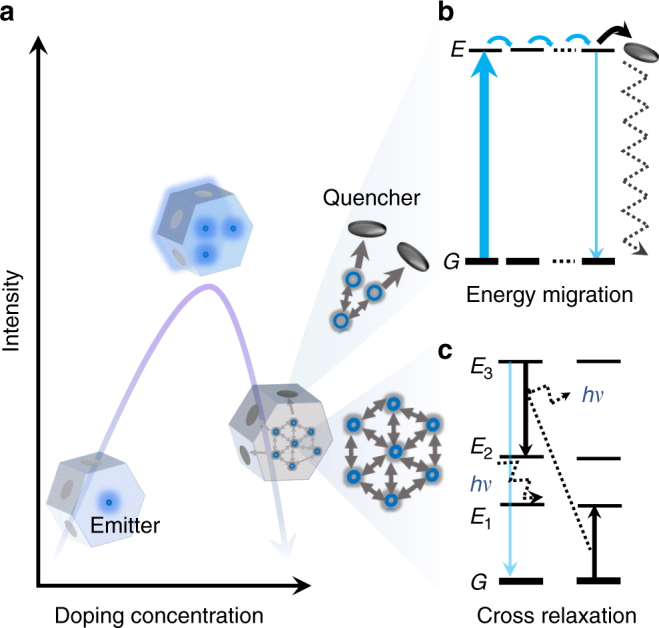
Concentration quenching in upconversion nanoparticles. **a** Increasing the doping concentration of dopant ions in the nanoparticles increases the number of photon sensitizers and emitters, shortens the distance from sensitizer to activator, and hence enhances the emission brightness, but surpassing a concentration threshold could make the cascade energy transfer process less effective, as the concentration quenching dominates with high levels of dopants. In a highly doped system, the concentration quenching is likely to be induced by: **b** non-radiative energy migration to surface quenchers and **c** cross-relaxation non-radiative energy loss. The term *hv* represents the phonon energy

The limitation set by the threshold of concentration quenching becomes a real problem for nanoscale luminescent materials (Figure 1a). As illustrated in Figure 1b, c, the general cause follows that high-doping concentration (shorter distance) leads to increased occurrence of energy transfer process between the dopants^5,7^. The excited-state electrons can be quickly short-circuited to the surface of nanomaterials, where a relatively large number of quenchers exist. Therefore, a dramatic decrease in luminescence intensity is observed. More specifically, the high-doping concentration facilitates both the energy migration of excited levels (typically within the sensitizer-sensitizer network) to the surface quenchers (Figure 1b)^15,24,25^ and the inter-dopant (typically between activators) cross-relaxation that causes emission intensity loss each time^7,13^ (Figure 1c).

To avoid the quenching of luminescence, conventionally, the doping level has been kept relatively low to ensure a sizable separation between the dopants to prevent parasitic interaction. Accordingly, the critical distance (Förster critical distance) is typically in the range of 2−6 nm^26^, meaning that the doping range should remain below 10^−3^ M for organic dye-doped SiO_2_^23^ and 10^−2^ M for UCNPs (Box 1)^13^. For an efficient upconversion to proceed, the relatively low concentrations of sensitizers (typically around 20 mol %) and activators (below 2 mol %) are generally used in the hexagonal-phase alkaline rare-earth fluoride nanocrystal, β-AREF_4_, that is known as one of the most efficient host material for upconversion. Low-doping concentration is the key roadblock to yield smaller and brighter luminescent nanomaterials^27–29^, which requires a canonical approach to optimizing the composition and chemical architecture of nanoparticles as well as photoexcitation schemes^4,6^.

### Box 1. The mechanism of concentration quenching

The notorious photophysical phenomenon of concentration quenching is frequently observed in solutions containing a high concentration of organic dyes, typically in the range of 10^−3^–10^−2^ M^7,9,19,20^. The leading factors for concentration quenching involve Förster resonance energy transfer (FRET) and Dexter electron transfer (DET). FRET is based on classical dipole–dipole interactions between the transition dipoles of the donor (D) and acceptor (A). The rate of the energy transfer decreases with the D–A distance, *R*, falling off at a rate of 1/*R*^6^. DET is a short-range phenomenon that falls off exponentially with distance (proportional to e^−*kR*^, where *k* is a constant that depends on the inverse of the van der Waals radius of the atom) and depends on spatial overlap of donor and quencher molecular orbitals^16^. The concentration quenching for phosphors is expressed by the rate constant, *k*_CQ_, determined from the equation for *η*_PL_:^30^

1 $$k = \frac{1}{{T_{{\mathrm{PL}}}}}\left( {\frac{{R_0}}{R}} \right)^6$$

2 $$R_0 = \root {6} \of {{\frac{{9000c^4\ln 10x^2\eta _{{\mathrm{PL}}}}}{{128\pi ^5n^4N_A}}{\int} {f_{\mathrm{D}}\left( v \right)\varepsilon _{\mathrm{A}}\left( v \right)\frac{{{\mathrm{d}}v}}{{v^4}}} }}$$

3 $$\eta _{{\mathrm{PL}}} = \eta _{{\mathrm{et}}}\eta _{{\mathrm{isc}}}\frac{{k_{{\mathrm{ph}}}}}{{k_{{\mathrm{ph}}} + k_{{\mathrm{nr}}} + k_{{\mathrm{CQ}}}}}$$

where *T*_PL_ is the intrinsic radiative decay time of the D, *R* is the distance between D–A and *R*_0_ is the Förster radius at which the energy transfer efficiency between D and A falls to 50%, *η*_et_ is the energy transfer efficiency, *η*_isc_ is the intersystem crossing efficiency, *k*_ph_ and *k*_nr_ are the rate constants of radiative and non-radiative decay, respectively.

When this analogy extends to an inorganic system, such as UCNPs, the concentration quenching denotes the emission intensity decrease phenomenon as the dopant concentration is too high. Typically, the UCNPs contain two types of lanthanide dopants, that is, the sensitizer (D) and activator/emitter (A)^2,7^. Though some singly doped (for example Er^3+^) particles can generate upconversion, researchers prefer to employ a sensitizer (for example Yb^3+^) to enlarge the absorbance in the NIR for enhanced upconversion luminescence. The process of the energy extraction from a sensitizer to an acceptor usually takes place via a non-radiative exchange (DET) or a multipolar interaction (FRET)^31^.

Most of the lanthanides have plentiful excited states, which show a high possibility to couple with one another through multipolar interactions with matching energy gaps, known as cross-relaxation (Fig. 1c)^7^. But this kind of coupling effect only occurs when the doping concentration is above a certain threshold. Because the 4*f*–4*f* transitions are symmetry forbidden according to the Laporte selection rule, the consequences are low intensity and long-decay time^32,33^. This cross-relaxation is primarily responsible for concentration quenching because neighbour ions, one in the excited state and the other in the ground state, non-radiatively exchange energy generally followed by phonon relaxation. The cross-relaxation process is evidenced by shorter lifetimes and decreased luminescence intensity.

Considering the energy transfer between two, or a network of, identical ions (for example, Yb^3+^ ions) in a concentrated system, the excited electrons will hop among the network. Such a process can quickly bring the excitation energy far away from the initial excited sensitizer, known as energy migration (Fig. 1b). In some specific systems, energy migration may be advantageous because it enables the upconversion from Eu^3+^ or Tb^3+^ emitters infused with an Gd^3+^ ion network^34^, or for Nd^3+^-based sensitization with the assistance of an Yb^3+^ ion network^35^. However, these systems would short-circuit the excitation energy to quenchers. 

### Emerging strategies to overcome concentration quenching

Recently, great efforts have been made to overcome concentration quenching in luminescent nanoparticles, particularly from the upconversion research community. Various approaches have been developed to alleviate the threshold of concentration quenching in both homogeneously doped nanocrystals and heterogeneously doped core@shell nanocrystals. Box 2 summarizes four strategies that have proven effective for homogeneously doped nanocrystals. One of the most commonly used schemes is to passivate the particle’s surface with an inert shell (for example, NaYF_4_, NaGdF_4_, NaLuF_4_ or CaF_2_), which can block the pathway of energy migration to surface quenchers^15,25,36^. The second strategy is to irradiate the particles with a high-energy flux that is sufficient for the activation of all dopant ions^37^. The third strategy is to choose a host crystal featuring a large unit cell to keep the D–A distance large enough even for stoichiometric compositions^24,38^. And the last strategy is to improve the doping uniformity in the host nanomaterials^36,39^, which minimizes the segregation of ions and thus prevents local concentration quenching.

Using wet-chemical synthesis methods developed over the past decade, it becomes possible to accurately control both the number and spatial distribution of dopants. This paves the way for a more efficient synthesis of heterogeneously doped core@multishell nanocrystals, which allows for the optimization of doping concentrations in each layer and selective isolation of different lanthanide ions to lower the probability of deleterious cross-relaxation. For example, Pilch et al. systematically studied a series of core@shell UCNPs to evaluate the effect of core@shell architecture on sensitizer and activator ions^40^; by separating sensitizers and emitters through the use of multilayer core@shell nanostructures, the concentration quenching threshold of Er^3+^ was lifted from 2 to 5%^41^.

### Box 2. Main strategies to overcome concentration quenching

**a** Coating an inert shell: Inert shell passivation on a low-doping luminescent core is a common strategy to avoid surface quenching by shielding the luminescent core from surface quenchers. This strategy further helps highly doped UCNPs by preventing the migration of sensitized photon energy to the surface quenchers, providing a convenient solution for detrimental quenching effects^15,25,36,42^.

**b** Increasing excitation power density: In the case of low irradiance, concentration quenching happens in highly doped UCNPs because too many nearby ground-state ions will easily take away the energy of excited-state ions. By supplying a high irradiance, either by using a high-power laser or focusing the excitation beam, a sufficient amount of excitation photon flux will be supplied to the large number of highly doped ions, and the majority of them will be at excited (intermediate) states, which reduces the number of detrimental ground-state ions. This strategy has proven highly effective in alleviating the thresholds of concentration quenching in UCNPs involving Tm^3+^ or Er^3+^ as emitters^17,18^. Benz et al. also described a rate equation model, which shows that the increased luminescence intensity for highly doped nanocrystals at a high irradiance is due to the enlarged distance between excited ions and ground-state ions^37^.

**c** Choosing a host nanocrystal with a large unit cell size^24,38^: The minimum distance between two dopants in a nanocrystal is determined by the size of the unit cell. Taking the β-NaYF_4_ crystalline structure as an example, the average distance between a sensitizer and an acceptor can be approximated using the following equation: $d_{{{\beta}} - {\mathrm{NaYF}}_4} = \left( {\frac{{a^2c\sqrt 3 /2}}{{1.5(x + y)}}} \right)^{1/3}$, which is evolved from the known lattice parameters when ignoring the lattice distortion caused by doping^31^. The parameters *x* and *y* represent the doping concentrations of the sensitizer and acceptor, respectively, while *a* and *c* are the lattice parameters of the hexagonal unit cells.

**d** Improving the homogeneous distribution of dopants: A homogeneous distribution of dopants can avoid local concentration quenching. The layer-by-layer hot injection strategy is technically superior to conventional one-pot heating up synthesis procedures in achieving a high uniformity in the dopant distribution^43^. Precise control on the Angstrom scale of the dopant distribution may be achieved by managing the rate of precursor injection^43.^ 

### Homogeneously doped nanocrystals

**Fig. 2:**
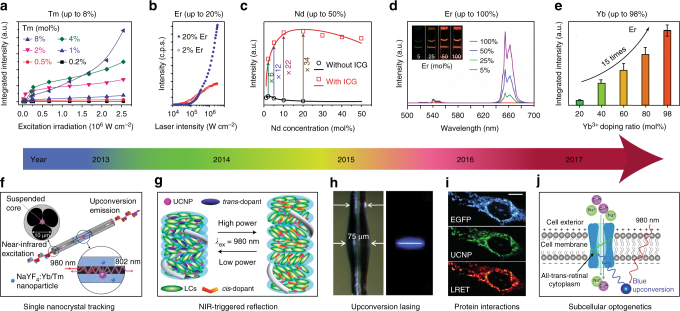
Selected milestones overcoming concentration quenching in homogeneously doped upconversion nanocrystals. **a** Integrated upconversion luminescence intensity as a function of excitation irradiance for a series of Tm^3+^-doped (0.2–8%) nanocrystals. Adapted from ref. ^17^. **b** Upconversion luminescence intensity of single 8 nm UCNPs with 20 and 2% Er^3+^, each with 20% Yb^3+^, plotted as a function of excitation intensity. Adapted from ref. ^18^. **c** Experimental results (black circle and red square) and theoretical modelling (black and red curves) of integrated upconversion luminescence intensities of a set of NaYF_4_:Nd^3+^ UCNPs with and without indocyanine green (ICG) sensitization. Adapted with permission from ref. ^45^ Copyright (2016) American Chemical Society. **d** Luminescence spectra of colloidal dispersion of NaYF_4_:*x*%Er@NaLuF_4_ nanocrystals (*x* = 5, 25, 50, 100); Inset: luminescence images of NaYF_4_:*x*%Er@NaLuF_4_ in cyclohexane excited with a 980 nm laser. Adapted with permission from ref. ^25^ Copyright (2017) American Chemical Society. **e** Integrated upconversion luminescence intensity of α-NaY_0.98-*x*_Yb_*x*_F_4_:2%Er@CaF_2_ (*x* = 0.2, 0.4, 0.6, 0.8, 0.98). Adapted with permission from ref. ^47^ Copyright (2017) Royal Society of Chemistry. **f** Schematic of the experimental configuration for capturing upconversion luminescence of NaYF_4_:Yb^3+^,Tm^3+^ nanocrystals using a suspended-core microstructured optical-fibre dip sensor. Adapted from ref. ^53^. **g** Upon irradiation by a NIR laser at the high-power density, the reflection wavelength of the photonic superstructure red-shifted, whereas its reverse process occurs upon irradiation by the same laser but with the lower-power density. Adapted with permission from ref. ^55^ Copyright (2014) American Chemical Society. **h** Photographs of a microresonator with and without optical excitation. Adapted from ref. ^56^. **i** UCNPs functionalized with a nanobody recognizing enhanced green fluorescent protein (EGFP) could rapidly and specifically target to EGFP-tagged fusion proteins in the mitochondrial outer membrane, and this protein interaction process could be detected by lanthanide resonance energy transfer (LRET) in living cells. Scale bar: 10 μm. Reproduced with permission from Drees et al.^57^ copyright John Wiley and Sons. **j** Schematic of channelrhodopsin-2 activated in HeLa cells by strong blue upconversion luminescence from NaYbF_4_:Tm^3+^@NaYF_4_ core@shell structure. Adapted with permission from ref. ^58^ Copyright (2017) American Chemical Society

Building brighter nanocrystals: Highly doped homogeneous UCNPs displaying exceptional brightness were first reported by Zhao and coworkers^17^. As shown in Figure 2a, an increase in the excitation irradiance from 1.6 × 10^4^ to 2.5 × 10^6^ W cm^−2^ enhances the overall luminescence intensity by a factor of 5.6, 71 and 1105 for 0.5, 4 and 8% Tm^3+^-doped nanocrystals, respectively^17^. The high brightness of these Tm^3+^-doped UCNPs allows the tracking of single nanoparticles in living cells through an optical microscope^44^. A similar trend was observed in highly Er^3+^-doped NaYF_4_:Yb^3+^ sub-10 nm nanocrystals (Figure 2b). As the excitation power increases, conventional UCNPs (2% Er^3+^) saturate in brightness while the highly doped UCNPs (20% Er^3+^) appear much brighter than the conventional UCNPs^18^. The optimal concentration for Nd^3+^ (conventionally around 1%) was increased to 20% with the sensitization of indocyanine green (ICG), resulting in about a tenfold brightness enhancement (Figure 2c)^45^. Encouragingly, the upconversion emission from a series of NaYF_4_:*x*%Er^3+^@NaLuF_4_ nanocrystals clearly showed high brightness at high-doping concentrations of Er^3+^, with NaErF_4_@NaLuF_4_ (100% doping) being the brightest (Fig. 2d)^25^.

The optimal concentration of Yb^3+^ sensitizer ions has also been pushed to its limit. Chen et al. reported that highly Yb^3+^-doped sub-10 nm cubic phase (α-) NaYF_4_:Yb^3+^,Tm^3+^ nanocrystals display a 43 times emission enhancement at NIR wavelengths compared to the conventional 20% Yb^3+^-doped one^46^. With an inert shell coating, the α-NaYbF_4_:2%Er^3+^@CaF_2_ UCNPs display a 15-fold enhancement in visible light emission (Figure 2e)^47^. Using an inert CaF_2_ shell, heavy doping of Yb^3+^ ions in the ultra-small α-NaYF_4_:Yb^3+^,Tm^3+^ and α-NaYbF_4_:Tb^3^^+^ nanoparticles was found to dramatically enhance the upconversion emission for high-contrast bioimaging^48–51^. With a NaYF_4_ inert shell, the optimal concentration of Yb^3+^ sensitizer ions was quantitatively examined by characterizing β-phase UCNPs at the single-particle level^52^.

Emerging applications: With exceptional brightness and associated optical properties, a range of applications have emerged. For example, highly Tm^3+^-doped UCNPs enable single nanoparticle sensitivity using a suspended-core microstructured optical-fibre dip sensor (Fig. 2f)^17,53^. The diverging brightness trends enable the optical encoding application by varying the excitation intensities^18^. More recently, Li et al. employed living yeast as a natural bio-microlens to concentrate the excitation energy of light and to enhance upconversion luminescence, suggesting a new way of detection of cells^54^.

High efficiency of upconversion emission from the NIR to the UV was achieved by high Yb^3+^ doping in NaGdF_4_:70%Yb^3+^,1%Tm^3+^@NaGdF_4_ nanostructures, and by varying the NIR excitation power density the reversible dynamic red, green and blue reflections of superstructure in a single thin film was demonstrated (Figure 2g)^55^. Through confined energy migration, an efficient five-photon upconverted UV emission of Tm^3+^ has been demonstrated in a NaYbF_4_ host without concentration quenching, and the large amount of spontaneous upconversion emission provides sufficient gain in a micron-sized cavity to generate stimulated lasing emissions in deep-ultraviolet (around 311 nm) wavelength (Figure 2h)^56^.

Using an increased excitation power density and highly Er^3+^-doped NaYF_4_:20%Yb^3+^,20%Er^3+^@NaYF_4_ UCNP, Drees et al. reported that resonance energy transfer has been enhanced by more than two orders of magnitude compared to that of standard NaYF_4_:20%Yb^3+^,2%Er^3+^@NaYF_4_ particles being excited at a low-power density^57^. After conjugation with anti-GFP nanobodies, the formed UCNP nanoprobe was used to target GFP fusion proteins inside living cells via a blue upconversion luminescence-induced resonance energy transfer process (Figure 2i)^57^. More recently, Pliss et al. reported that NaYbF_4_:0.5%Tm^3+^@NaYF_4_ UCNPs emit six times higher blue emission, compared to typical NaYF_4_:30%Yb^3+^,0.5%Tm^3+^@NaYF_4_ UCNPs, for effective optogenetic activation using NIR light (Figure 2j)^58^.

### Heterogeneously doped nanocrystals

**Fig. 3:**
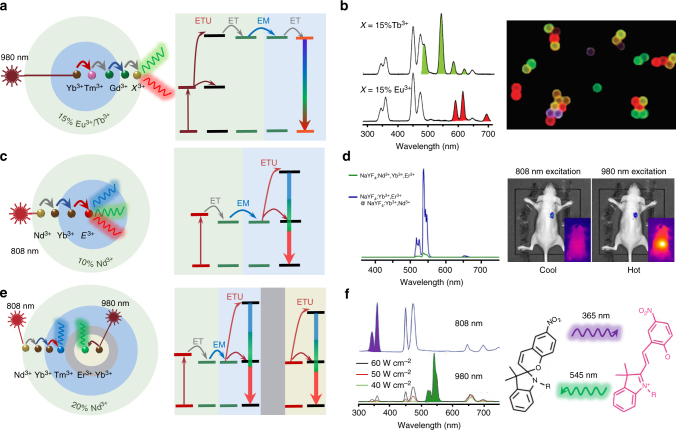
Heterogeneously doped core@shell upconversion nanoparticles to overcome concentration quenching. **a** The schematic of a core@shell design with energy migration at the emission part^34^. **b** Upconversion emission spectra with tunable wavelengths attributed to a prescribed Yb^3+^→Tm^3+^→Gd^3+^→Eu^3+^/Tb^3+^ energy cascade across the core@shell interface, and luminescence micrograph of polystyrene beads tagged with core@shell nanoparticles. Adapted from ref. ^34^. **c** Schematic of the core@shell design with energy migration at the excitation part, *E*^3+^ = Er^3+^, Tm^3+^, or Ho^3+^ ^35^. **d** Upconversion emission spectra of NaYF_4_:Yb^3+^,Er^3+^@NaYF_4_:Yb^3+^,Nd^3+^ core@shell and homogeneous-doped nanoparticles, and the in vivo imaging of the core@shell nanoparticles under 808 nm and 980 nm excitation, respectively. Adapted with permission from ref. ^35^ Copyright (2013) American Chemical Society. **e** Schematic of doping location control in the core@shell design with an energy transfer blocking layer^80^. **f** Core@multishell UCNPs (NaYF_4_:Yb^3+^,Nd^3+^,Tm^3+^@NaYF_4_@NaYF_4_:Yb^3+^,Er^3+^) with orthogonal emissions under irradiation at 800 and 980 nm, and the orthogonal emissions were employed for reversible isomerization of spiropyran derivatives. Adapted with permissions from Lai et al.^80^ Copyright John Wiley and Sons

The precision in controlled growth has resulted in a library of intentional heterogeneously doped core@shell UCNPs^59,60^. The doping concentrations in multilayers of nanostructure can be optimized to satisfy the requirements of a particular application, for example, according to either excitation conditions^16,35,61,62^ and/or desirable emission wavelengths^34,63,64^, to produce a high-performance energy-migration-mediated upconversion process (Figure 3)^16^ or efficient light-to-heat conversion^65^.

Controlled energy migration: As shown in Figure 3a, the fine-tuning of upconversion emission colours through energy migration has been first demonstrated using a Gd^3+^ sublattice structure as an efficient energy transfer bridge across the core@shell interface. As shown in Figure 3b, upconversion emission with tunable wavelengths and lifetimes has been realized via a prescribed energy cascade of Yb^3+^ → Tm^3+^ → Gd^3+^ → lanthanide activators (for example, Tb^3+^, Eu^3+^, Dy^3+^ and Sm^3+^)^34^. Note that the efficiency of energy-migration-mediated upconversion emission of Tb^3+^, Eu^3+^ or Dy^3+^ at high-doping concentrations is more than two orders of magnitude of that from a Yb^3+^-sensitized cooperative energy transfer system^32^. Efficient photon upconversion has also been demonstrated through the heterogeneous core@shell nanostructure of NaYbF_4_:Gd^3+^,Tm^3+^@NaGdF_4_@CaF_2_:Ce^3+^ with a high-doping concentration of Ce^3+^ in the shell layer^66^. A CaF_2_ host has been employed to reduce the 4*f*–5*d* excitation frequency of Ce^3+^ to match the energy level of Gd^3+^. Zhang et al. have fabricated NaGdF_4_:Yb^3+^,Tm^3+^,Er^3+^@NaGdF_4_:Eu^3+^@NaYF_4_ incorporated with RGB-emitting lanthanide ions at high concentration, and generated high-brightness white light across the whole visible spectrum^67^. Notably, apart from the lanthanide ions, organic dyes tethered on the surface of NaGdF_4_:Yb^3+^,Tm^3+^@NaGdF_4_ nanocrystals can accept the sensitized UV energy through the Gd^3+^-mediated energy transfer, which can dramatically improve the sensitivity in FRET-limited measurements^68^. Apart from Gd^3+^ as the energy mediation ions, due to a large energy gap (Δ*E* *>* 5 *hν*) between respective levels^69,70^, Mn^2+^ and Tb^3+^ ions also show a similar function for energy migration from the core to shell when doped by high concentration of activators.

Exciting UCNPs at 980 nm, through the transparent biological window (650–1350 nm), offers higher photo-biocompatibility and allows deeper tissue penetration than that achievable at 532 nm, as living cells can withstand around 3000 times more intensity at 980 nm than at 532 nm visible excitation^71^. Considering the fact that water absorbs around 20 times more excitation light at 980 nm than at 800 nm, researchers have further designed core@shell UCNPs by shifting the excitation wavelength to around 800 nm^35^. The key is to use Nd^3+^ ions as the sensitizer to absorb 800 nm photons. Because the absorption cross-section of Nd^3+^ ions at 800 nm is 25-fold larger than that of Yb^3+^ ions at 980 nm, Nd^3+^-doped UCNPs display brighter upconversion emissions and negligible overheating effects^72^. Since homogeneously co-doping high concentration of Nd^3+^ ions and activators will quench the overall upconversion emission, owing to the deleterious back energy transfer from activators to Nd^3+^ ions, the doping concentration of Nd^3+^ has been limited to below 1%. To overcome this threshold, an energy migration system has been employed to separate Nd^3+^ ions and activators (Figure 3c). Yan et al. first demonstrated high-concentration doping of Nd^3+^ in core@shell UCNPs that displayed a much enhanced upconversion luminescence relative to those homogeneously doped (Figure 3d). Using these Nd^3+^-sensitized UCNPs, the authors further demonstrated superior imaging performance for in vivo imaging without the issue of tissue overheating (Figure 3d)^35^. However, further studies are still needed to investigate the penetration depth trade-off of using 800 nm excitation light, since the amount of light scattering increases in proportion to the fourth power of the frequency of the light. To this regard, a future research direction is to shift the excitation wavelength from the first NIR optical window (650–1000 nm) to the second NIR spectral window (1100–1350 nm), which is ideal for deep-tissue imaging because of reduced water absorption and light scattering^73^.

To optimize the doping concentration in core@shell structures, Xie et al. have found that the design of NaYF_4_:20%Yb^3+^,0.5%Tm^3+^,1%Nd^3+^@NaYF_4_:20%Nd^3+^ results in upconversion emission around seven times stronger than that of NaYF_4_:Nd^3+^,Yb^3+^,Tm^3+^@NaYF_4_^61^. To further reduce the cross-relaxation and back energy transfer from activators to Nd^3+^ sensitizers, Zhong et al. reported a nanostructure design in the form of NaYF_4_:Yb^3+^,Er^3+^@NaYF_4_:Yb^3+^@NaNdF_4_:Yb^3+^, in which the intermediate NaYF_4_:Yb^3+^ shell separates Er^3+^ activators from Nd^3+^ primary sensitizers^74^. With the doping concentration of Nd^3+^ being pushed to 90%, there is eight times more upconversion luminescence produced, compared to the NaYF_4_:Yb^3+^,Er^3+^@NaYF_4_:Nd^3+^ structure. Encouragingly, a more sophisticated doping pattern of NaYF_4_:2%Er^3+^,30%Yb^3+^@NaYF_4_:20%Yb^3+^@NaNdF_4_:10%Yb^3+^, with fine-tuning of Yb^3+^ concentrations at different layers, was found to facilitate more efficient energy transfer process of (Nd^3+^ → Yb^3+^) → (Yb^3+^) → (Yb^3+^ → Er^3+^) at higher Nd^3+^ doping concentration. This design further reduces the requirement in the excitation power, so that upconversion luminescence has been observed even under a 740 nm LED^75^. A similar energy migration strategy has been proposed to get cooperative emission from Tb^3+^ ions, which demonstrated a tenfold upconversion enhancement under 800 nm photoexcitation of Nd^3+^ ions as compared to Yb^3+^ sensitization.^76^ The ratiometric Nd^3+^ → Yb^3+^ and Yb^3+^ → Er^3+^ energy transfer processes in the core@shell nanocrystals have also been used for temperature sensing in two different temperature ranges^77^. More detailed and systematic studies of the concentrations of both the primary sensitizer and secondary sensitizers will improve the efficiency of energy cascade.

Combining strategies presented above (Figure 3a, c), upconversion tuning with high-doping concentrations can be achieved by either Gd^3+^-mediated energy migration or Yb^3+^-mediated absorption/migration. For example, the core@multishell structure of NaYbF_4_:50%Nd^3+^@NaGdF_4_:Yb^3+^,Tm^3+^@NaGdF_4_:*A* (*A*: activator Eu^3+^, Tb^3+^, or Dy^3+^) has been utilized to initiate the energy migration from Gd^3+^ to the activators with high concentration^78^. In this system, the Nd^3+^-sensitized UCNPs displayed emissions spanning from the UV to the visible region with high efficiency through a single wavelength excitation at 808 nm. More recently, Liu et al. designed a multilayer nanoparticle for simultaneously displaying short- and long-lived upconversion emission with high concentrations of different dopants at different layers, making multilevel anti-counterfeiting possible at a single-particle level^79^.

Blocking layer separation: In addition to the strategies of tuning excitation and emission properties, the control over doping location with a blocking layer (Figure 3e) has been demonstrated for orthogonal emissions^78,80,81^, spectral/lifetime multiplexing^82^, upconverting/downshifting^83^, multimode imaging^84^, and multi-optical functions in single particles^65^. For example, reversible isomerization of spiropyran derivatives has been achieved by the orthogonal emissions of core@multishell UCNPs with a high-doping concentration of Nd^3+^ under irradiation at 800 and 980 nm (Figure 3f)^80^. A similar idea was used to efficiently trigger a reversible photocyclization of the chiral diarylethene molecular switch by the UV and visible luminescence from core@multishell UCNPs with dual wavelength NIR light transduction properties^85^. The emission colours of these Ho^3+^/Tm^3+^ co-doped NaGdF_4_:Yb^3+^ UCNPs can be tuned by changing the laser power density or temperature, due to the different spectral responses^86^. By design and synthesis of NaGdF_4_:Nd^3+^@NaYF_4_@NaGdF_4_:Nd^3+^,Yb^3+^,Er^3+^@NaYF_4_ nanoparticles, both upconversion and downshifting luminescence, sensitized by highly doped Nd^3+^, can be achieved without cross interference^83^. Moreover, excited Nd^3+^ ions can transfer energy to other lanthanide ions and result in tunable downshifting emission. For instance, co-doping Yb^3+^ with Nd^3+^ at high concentrations would give an intense NIR emission centred around 980 nm due to the efficient energy transfer from Nd^3+^ to Yb^3+^^87^. The spectral and lifetime characteristics can correlate orthogonally with excitation by constructing noninterfering luminescent regions in a nanoparticle, which enables the multiplexed fingerprint and time-gated luminescent imaging in both spectral and lifetime dimensions^82^. More recently, Marciniak et al. have demonstrated the heterogeneous doping of Nd^3+^ ions with different concentrations in different parts of NaNdF_4_@NaYF_4_@NaYF_4_:1%Nd^3+^ nanoparticles to achieve three optical functions, namely efficient (*η* > 72%) light-to-heat conversion, bright NIR emission and relatively sensitive (*S*_R_ > 0.1% K^−1^) localized temperature quantification^65^. The undoped NaYF_4_ intermediate shell enables the separation of the 1% Nd^3+^-doped outer shell (for efficient Stokes emission) from 100% Nd^3+^-doped core (for cross-relaxation based efficient light-to-heat conversion).

### Emerging applications enabled by cross-relaxation

Cross-relaxation has often been perceived as being deleterious, but new research shows that cross-relaxation can render many unique properties, such as single-band emission^88,89^, energy looping^73^, tunable colour/lifetime^63,90^, enhanced downshifting emissions^91^ and photo-avalanche effect for amplified-stimulated emission^1,92^.

**Fig. 4:**
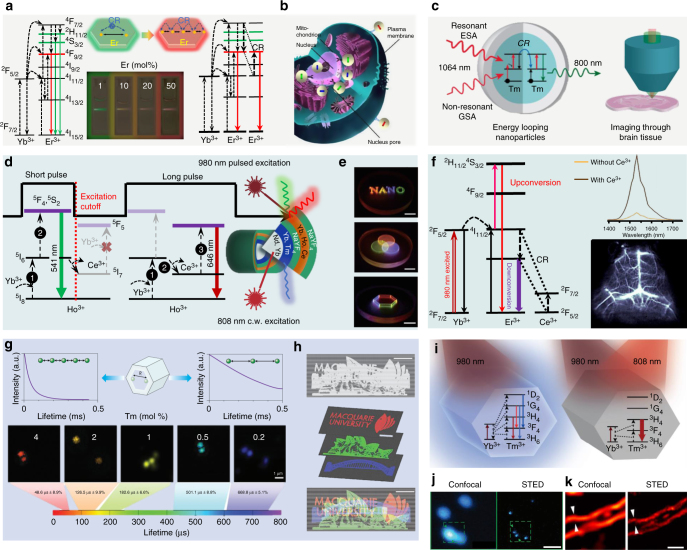
Cross-relaxation-enabled nanotechnology using highly doped upconversion nanocrystals. **a** Upconversion mechanisms of Er^3+^ at low- and high-doping levels and the fluorescence photographs of NaYbF_4_:Er^3+^ UCNPs with different concentrations of the activator, the cross-relaxation effect induces pure red emission with increasing Er^3+^ concentration. Adapted with permission from ref. ^89^ Copyright (2014) American Chemical Society. **b** Application of single-band upconversion nanoprobes for multiplexed in situ molecular mapping of cancer biomarkers. Adapted from ref. ^93^. **c** Core@shell design and energy-looping mechanism in highly Tm^3+^-doped NaYF_4_, and their application for deep-tissue brain imaging. Reproduced with permission from ref. ^73^ Copyright (2016) American Chemical Society. **d** Design and mechanism of NaYF_4_-based core@shell nanocrystals capable of emitting tunable colours through the combined use of a continuous wave 808 nm laser and a 980 nm laser with short or long pulse. Adapted from ref. ^63^. **e** Its application in 3D and full-colour display systems with high-spatial resolution and locally addressable colour gamut, scale bars represent 1 cm. Adapted from ref. ^63^. **f** Simplified energy-level diagrams depicting the energy transfer between Yb^3+^, Er^3+^, and Ce^3+^ ions, downshifting luminescence spectra of the NaYF_4_:Yb^3+^,Er^3+^ UCNPs with and without Ce^3+^ doping and their application for cerebral vascular image through the second NIR window. Adapted from ref. ^91^. **g** Lifetime tuning scheme and time-resolved confocal images of NaYF_4_:Yb^3+^,Tm^3+^ UCNPs with increasing concentration of Tm^3+^, scale bar represents 1 μm. Adapted from refs. ^90,101^. **h** Their application in lifetime-encoded document security, scale bars represent 5 mm. Adapted from ref. ^90^. **i** Energy-level diagrams of highly Tm^3+^-doped UCNPs under 980 nm and/or 808 nm illumination. Adapted from ref. ^1^. **j** Confocal versus STED super-resolution images of the 40 nm 8% Tm^3+^-doped UCNPs, scale bar represents 500 nm. Adapted from ref. ^1^. **k** Confocal versus STED super-resolution images of cellular cytoskeleton labelled with antibody-conjugated 11.8 nm NaGdF_4_:18%Yb^3+^,10%Tm^3+^ nanocrystals, scale bar represents 1 μm. Adapted from ref. ^92^

Single-band emission: High-throughput molecular profiling requires optical multiplexing of single-band emission probes to target multiple analytes without crosstalk (Figure 4b), but each lanthanide ion emitter in an UCNP has multiple energy levels with multiple emission peaks^93^. Cross-relaxation by high-doping concentration has been used to quench the unwanted emission bands to yield single-band emission^94,95^. Chan et al. used combinatorial screening of multiple doped NaYF_4_ nanocrystals to identify a series of doubly and triply doped nanoparticles with pure emission spectra at various visible wavelengths^96^. Approaching 100% red emission output has been reported by Wei et al. using highly doped activators, where the cross-relaxation effect dominates and quenches the green or blue emissions (Figure 4a)^89^. This strategy was successful in achieving pure red 696 or 660 nm upconversion emission as well as precisely tuning upconversion colours to study the underlying upconversion mechanisms.

More recently, Chen et al. presented a new class of β-NaErF_4_:0.5%Tm^3+^@NaYF_4_ nanocrystals with bright red upconversion luminescence through high concentration Er^3+^-based host sensitization, in which Tm^3+^ ions are employed to trap excitation energy and to minimize the luminescence quenching effect^97^. Introducing high concentrations of Ce^3+^ into NaYF_4_:Yb^3+^/Ho^3+^ or NaYF_4_:40%Gd^3+^ have greatly enhanced the red-to-green upconversion emission ratio of Ho^3+^ through effective cross-relaxation between Ce^3+^ and Ho^3+^^98,99^. Similarly, combining the strategy presented in Figure 3c, single-band red upconversion luminescence of Ho^3+^ has been achieved under 808 nm excitation from NaGdF_4_:Yb^3+^,Ho^3+^,Ce^3+^@NaYF_4_:Yb^3+^,Nd^3+^ core@shell nanoparticles with the shell layer highly doped with Nd^3+^ (around 10%)^88^. Also, doping a high concentration of Mn^2+^ into NaYF_4_:Yb^3+^,Er^3+^ nanocrystals has resulted in pure single-band red upconversion emission via an efficient energy transfer between Mn^2+^ and Er^3+^ ^95^. Levy et al. used an energy-looping mechanism to non-resonantly excite upconversion in highly Tm^3+^-doped NaYF_4_:Tm^3+^ nanoparticles with 1064 nm light for deep-tissue imaging^73^. In this work, as illustrated in Figure 4c, one Tm^3+^ ion can cross-relax by donating energy partially to a second Tm^3+^ ion in its ground state, resulting in two Tm^3+^ ions in their intermediate ^3^F_4_ state to enable efficient excited-state absorption at 1064 nm and emit 800 nm emissions^73^.

Full-colour/lifetime tuning: The energy transfer between the dopant ions in a core@shell nanostructure has also been found to be controllable by adjusting the pulse duration of the excitation laser (Figure 4d, e)^63^. By increasing the pulse duration from 0.2 to 6 ms (at 980 nm), the intensity ratio of green-to-red emission from the shell of NaYF_4_:Yb^3+^,Ho^3+^,Ce^3+^ with a high concentration of Ce^3+^ can be continuously modulated. The energy transfer from Ho^3+^ to Ce^3+^ by a cross-relaxation process ^5^I_6_(Ho^3+^) + ^2^F_5/2_(Ce^3+^) → ^5^I_7_(Ho^3+^) + ^2^F_7/2_(Ce^3+^) is only allowed under a long pulse excitation, while the transition from ^5^I_6_(Ho^3+^) to higher levels of ^5^F_4_, ^5^S_2_ prevails over the above cross-relaxation process involving Ce^3+^ by a short-pulse excitation. This judicious design has further generated pure blue upconversion emission by pumping at 800 nm by Nd^3+^ → Yb^3+^ → Tm^3+^ with a high concentration of Nd^3+^^63^. Cross-relaxation has been employed for enhancing the downshifting emission between 1500 and 1700 nm for high-spatial resolution and deep-tissue penetration of photons for cerebral vascular image in the second NIR window^91^. Facilitated by the high Ce^3+^-doping concentration, the Er^3+ 4^I_13/2_ level is significantly populated through the accelerated non-radiative relaxation of Er^3+ 4^I_11/2_ → ^4^I_13/2_ (Figure 4f), resulting in a ninefold enhancement of the downshifting 1550 nm luminescence of NaYbF_4_:2%Er^3+^,2%Ce^3+^@NaYF_4_ nanoparticles.

Apart from colour tuning, luminescence decay lifetimes form another set of optical signatures^79,82,90,100^. Manipulating the degree of cross-relaxation by different Tm^3+^-doping concentrations can create a large range of lifetimes from 25.6 to 662.4 μs in the blue emission band, forming a library of lifetime-tunable τ-dots for optical multiplexing (Figure 4g)^90,101^. Such an optical signature can be used as barcoding for security applications, and only a properly designed time-resolved detector can decode such a set of diverse time-domain optical barcodes. As demonstrated in Figure 4h, the ability to resolve superimposed lifetime-encoded images suggests a new way of optical data storage with high densities and fast data readout rates.

Super-resolution imaging: Another intriguing example is nanoscopic imaging using highly Tm^3+^-doped UCNP as an effective stimulated emission depletion (STED) probe. The advent of super-resolution microscopy, such as STED fluorescence microscopy, has revolutionized biological fluorescence microscopy^102,103^, but STED requires extremely high-power laser densities and specialized fluorescent labels to achieve super-resolution imaging. Using the cross-relaxation effect, Liu et al. discovered a photon avalanche effect that facilitates the establishment of population inversion within a single highly Tm^3+^-doped UCNP (Figure 4i)^1^. This enables sub-30 nm optical super-resolution imaging with a STED beam density two orders of magnitude lower than that used on fluorescent dyes (Figure 4j)^1^. This effect has only been found in highly doped UCNPs because the cross-relaxation process, dominated at a high Tm^3+^-doping concentration, can trigger a photon avalanche to establish a population inversion between metastable and ground levels. In that respect, upon 808 nm beam depletion amplified-stimulated emission is realized, resulting in a higher-depletion efficiency and thus a reduced saturation intensity. Using this new mode of upconversion nanoscopy, Zhan et al. have reported super-resolution imaging of cell cytoskeleton with 11.8 nm NaGdF_4_:18%Yb^3+^,10%Tm^3+^ UCNPs (Figure 4k)^92^. Other schemes, based on Pr^3+^ or Er^3+^ doped UCNPs, have also been explored for super-resolution nanoscopic applications^104,105^

### Perspective

One of the major challenges to transform upconversion nanotechnology into real-world applications is to enhance the brightness and emission efficiency of UCNPs^4^. This review summarizes the advances in the development of highly doped UCNPs and emerging applications by overcoming the concentration quenching effect or smart exploitation of unique features of highly doped nanomaterials. Notably, the unique optical properties arising from the range of layer-by-layer heterogeneously doped nanoparticles have attracted immense scientific and technological interests. The intentional doping of high concentration of lanthanide ions into different sections across a single UCNP has been explored to enhance the desirable optical properties as well as introducing multifunctionality. Thus far, only spherical core@shell structures have been studied to modulate the energy transfer, while further investigations of heterogeneous one-dimension structures, such as rods, plates and dumbbells, are still needed^60,106,107^. Controlled growth toward atomic precision is highly sought after for gaining a full understanding of the sophisticated energy transfer processes and for fine-tuning upconversion luminescence. For example, arranging high concentrations of dopants into a host nanocrystal along one direction could confine the direction of energy transfer, which may create new properties and enable novel applications going beyond the current isotropic 3D transfer processes.

The unique optical properties of highly doped UCNPs discussed above have largely impacted biological and biomedical fields, such as single-molecule sensing^18^, high-throughput multiplexed detection^79,82,90^ and super-resolution nanoscopy^1,92^. It is noteworthy that small-sized and bright UCNPs are indispensable to those applications. Owing to brightness issues, the majority of currently developed UCNPs are relatively large (around 20–50 nm). It has been challenging to design and fabricate highly doped sub-10 nm UCNPs with emission output comparable with that of quantum dots and organic dyes. To our delight, fine-tuning of the particle size below 10 nm was recently demonstrated for UCNP systems by homogeneous doping^108^ or at a high-doping concentrations^109^. Nonetheless, the fabrication of sub-10 nm UCNPs with heterogeneously doped core@shell structures remains a formidable challenge.

The surface molecules not only play an essential role in the controlled synthesis of nanomaterials, but they can also significantly alter the nanomaterial’s luminescence properties with new effects^110,111^. Examples include the recent developments of dye-sensitized UCNPs^16,62,112,113^ and surface phonon enhanced UCNPs in a thermal field^111^. Normally, lanthanide-doped inorganic nanocrystals exhibit narrowband (FWHM around 20 nm) and low (10^−20^ cm^−2^) absorption coefficients. It is notable that organic dyes have more than 10 times broader absorption spectra and 10^3^–10^4^-fold higher absorption cross-sections than Yb^3+^ sensitizer ions commonly used in UCNPs^16,62,112,113^. Therefore, despite photostability issues, the organic–inorganic hybrid nanomaterials (for example, dye-sensitized upconversion nanosystems) offer various possibilities^16^. Utilizing the efficient energy transfer of cyanine derivatives anchored on the surface of NaYbF_4_:Tm^3+^@NaYF_4_:Nd^3+^ nanoparticles, Chen et al. demonstrated dye-sensitized upconversion^16^. Upon 800 nm excitation, a sequential energy transfer, dye → Nd^3+^ → Yb^3+^ → activators, has enabled the dye-sensitized nanoparticles to emit around 25 times stronger than canonical NaYF_4_:Yb^3+^,Tm^3+^@NaYF_4_ nanoparticles excited at 980 nm. Similarly, the common triplet energy transfer could occur between inorganic nanocrystals and the surface dyes^114–118^. While thermal quenching broadly limits the luminescence efficiency at high temperatures in optical materials, Zhou et al. report that the phonons at the surface of highly Yb^3+^-doped UCNPs could combat thermal quenching and significantly enhance the upconversion brightness, particularly for sub-10 nm nanocrystals^111^. We believe that these hybrid and heterogeneously doped nanomaterials have the potential in pushing the performance of UCNPs to a new level and imparting multifaceted photonic applications.
